# Integrating Large-Scale Data and RNA Technology to Protect Crops from Fungal Pathogens

**DOI:** 10.3389/fpls.2016.00631

**Published:** 2016-05-31

**Authors:** Ian J. Girard, Austein G. Mcloughlin, Teresa R. de Kievit, Dilantha W. G. Fernando, Mark F. Belmonte

**Affiliations:** ^1^Department of Biological Sciences, University of ManitobaWinnipeg, MB, Canada; ^2^Department of Microbiology, University of ManitobaWinnipeg, MB, Canada; ^3^Department of Plant Science, University of ManitobaWinnipeg, MB, Canada

**Keywords:** RNA-seq, fungi, plant diseases, laser microdissection, bioinformatics, RNAi

## Abstract

With a rapidly growing human population it is expected that plant science researchers and the agricultural community will need to increase food productivity using less arable land. This challenge is complicated by fungal pathogens and diseases, many of which can severely impact crop yield. Current measures to control fungal pathogens are either ineffective or have adverse effects on the agricultural enterprise. Thus, developing new strategies through research innovation to protect plants from pathogenic fungi is necessary to overcome these hurdles. RNA sequencing technologies are increasing our understanding of the underlying genes and gene regulatory networks mediating disease outcomes. The application of invigorating next generation sequencing strategies to study plant–pathogen interactions has and will provide unprecedented insight into the complex patterns of gene activity responsible for crop protection. However, questions remain about how biological processes in both the pathogen and the host are specified in space directly at the site of infection and over the infection period. The integration of cutting edge molecular and computational tools will provide plant scientists with the arsenal required to identify genes and molecules that play a role in plant protection. Large scale RNA sequence data can then be used to protect plants by targeting genes essential for pathogen viability in the production of stably transformed lines expressing RNA interference molecules, or through foliar applications of double stranded RNA.

## Introduction

The world’s population is expected to increase to nearly 10 billion people in the next 35 years (United Nations Department of Economic, and Social Affairs, Population Division, 2015). To meet the demands of a growing population, it is estimated we will need to increase the production of safe, healthy and just food by 60–110% over current rates without an increase in arable land (Tilman et al., 2011; Ray et al., 2013). With fungal pathogens capable of destroying 60% of all crops in a sever epidemic (Fisher et al., 2012), it is an immediate concern and a priority for plant science researchers, breeders, and growers to find new, innovative and translatable solutions to protect global food systems. Protecting crops from major fungal outbreaks is traditionally done by employing either lengthy crop rotation times, undesirable for many cash crop producers, or by the application of broad spectrum fungicides that can have adverse consequences for the environment (Podio et al., 2008), and limited usefulness due to development of resistance (Hirooka and Ishii, 2013). To overcome the negative impacts of disease on food production, plant science researchers are turning to modern cutting edge molecular techniques to uncover and understand the underlying genes and gene regulatory networks in host–pathogen interactions. A deep understanding of the biology behind the processes that drive either plant tolerance and resistance or susceptibility, is required for breeding new crops and implementing the next generation of pathogen control measures.

**FIGURE 1:**
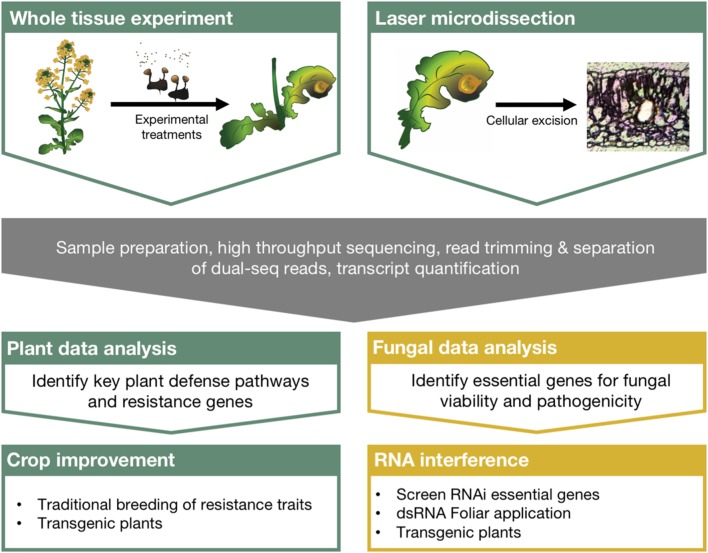
**Comprehensive strategy linking large scale RNA sequencing experiments with crop protection using RNA interference.** Dual sequencing and laser microdissection can overcome many of the limitations of traditional sequencing experiments and greatly improve our understanding of plant–fungal pathosystems. Robust bioinformatics strategies to identify critical regulators of plant defense and fungal pathogenesis can then be directly integrated with crop improvement strategies as well as RNA interference applications for crop protection from pathogenic fungi.

For example, global RNA profiling experiments are used to understand gene activity and can evaluate implicit changes in biological processes following the plant–fungal interaction. High throughput sequencing technologies have been available to the scientific community for over a decade now (Metzker, 2009), and more specialized techniques are being developed to investigate chromatin modifications, microRNAs, and RNA–protein interactions (Reuter et al., 2015). More recently, dual sequencing experiments, those that profile RNA from both the host and the pathogen, have been used to further our understanding of complex molecular interactions. Despite these advancements and the abundance of ‘big data’ generated to understand host–pathogen interactions, the bottleneck in understanding its genetic and biological relevance lies in the distillation process. Additionally, no clear link has been described between the scientific insights taken from these experiments and meaningful ways to protect crops. Here we discuss the integration of RNA sequencing to plant–fungal pathogen interaction studies, the technologies that will increase our resolution and understanding of the complex transcriptional circuitry regulating these interactions, and describe a direct path leading from these experiments to the protection of crops in the field (outlined in **Figure 1**).

## Dual Sequencing of Host–Pathogen Interactions

Thus the resulting sequence reads from dual-sequencing experiments, regardless of origin, contain a snapshot of the underlying transcriptional programs from both the host and pathogen. The reads that successfully map to the respective genomes can then be used to assess gene activity in the two species. A general dual sequencing experimental outline is described by Westermann et al. (2012), however, since its publication, the cost of sequencing experiments has gone down further, supporting the the accessibility of RNA-seq and opening new opportunities for the development of dual sequencing study systems.

Despite the advantages of dual-sequencing, relatively few studies investigate fungal–plant interactions using this approach. For example, *Septoria tritici*, one of the most economically important wheat pathogens, was shown to alter gene activity to marginalize wheat defenses during its biotrophic phase before transitioning to a necrotic lifestyle and causing plant disease (Yang et al., 2013). However, this study omits an investigation into the transcriptional changes during the infection process with an incompatible host, thus forgoing critical insights to plant immunity. Using this strategy, Kawahara et al. (2012) discovered a number of rice transcripts highly upregulated specifically during an incompatible interaction with the blast fungus *Magnaporthe oryzae*. Comparing the differences between resistant and tolerant lines of crop systems should help researchers discover key attenuations of the plant defense response, and provide answers into the genetic and molecular mechanisms underlying plant immunity.

Because of the diverse nature of economically important fungal–plant pathogens, researchers may not have access to reference genomes or transcriptome assemblies to aid in pathogen gene expression analysis. Previous RNA profiling techniques such as microarrays, are expensive to develop and require *a priori* knowledge of the organism (Wang et al., 2009). This limitation is easily overcome with RNA-seq experiments, wherein there exists well-established computational tools to generate transcriptome profiles from raw sequencing data (Grabherr et al., 2011; Martin and Wang, 2011; Ward et al., 2012). With this approach, Yazawa et al. (2013) identified putative transcription factors (TFs) and cell wall degrading enzymes expressed by *Bipolaris sorghicola* while a resistant line of its host, sorghum, activates putative WRKY TFs along with other defense-related genes. Likewise, this approach can be effectively used to study host–pathogen interactions in non-model hosts. For example, researchers studying sudden oak death used the available *Phytophthora ramorum* reference annotation to separate dual RNA-seq reads, and generated a reference transcriptome of its host (Hayden et al., 2014). These studies exemplify how effectively RNA-seq technology can be directly applied to translate model system data to help understand critically important fungal pathosystems.

In most cases, annotation tools developed from model systems to assign biological information to transcriptomic data are designed to be used independently on plants and fungi. Many tools and databases exist for plants, for example, *Arabidopsis*^1^, soybean^2^, rice^3^, maize, and many other grasses (Grant et al., 2010; Stein et al., 2014; Krishnakumar et al., 2015). However, only recently have comprehensive tools been available for fungi, with development of FungiFun2^4^ (Priebe et al., 2015). Network inference is another powerful tool to predict molecular interactions between hosts and pathogens by analyzing co-expressed gene sets and has been successfully used to discover interactions between immune cells and fungi in a mouse system (Tierney et al., 2012). Tools built specifically for dual RNA-seq experiments are in their infancy but promise to serve unique roles in unraveling gene regulatory networks in host–pathogen interactions (Schulze et al., 2015, 2016). Future expansion and development of these programs into general bioinformatics tools such as the Galaxy Project^5^, should proceed with a focus on building user-friendly interfaces in an open-access forum in order to maximize their utility within the plant science community.

## Laser Dissection of Host–Pathogen Interactions

One of the limitations of traditional RNA-seq experiments is that it evaluates the collective population of mRNAs from a complex multicellular tissue or organ system. This is particularly problematic for investigating early stages of infection where limited fungal biomass means few sequence reads relative to the host can be detected using traditional technology. In a recent dual sequencing experiment, Rudd et al. (2015) demonstrated that *Zymoseptoria tritici* modifies defense gene activity without significant nutrient acquisition from the host during early stages of infection before degrading and consuming host-derived nutrients during necrosis. However, less than 2% sequence reads from *Z. tritici* infected wheat mapped to the fungal genome at 4 days post inoculation, which increases to 40% at 14 days post inoculation. The limited sequencing depth directly results in limited quality of the RNA-seq data for early stages of infection, potentially obfuscating early events critical to pathogenesis. The result of any interactions between a fungal–pathogen and its plant host is specified at the cellular level, directly at the site of infection. Thus, the microscopic scale of these interactions is a major limiting factor on the quality of sequencing experiments as traditional protocols may dilute early signaling events and molecular responses will have faded beyond detection limits by the majority of transcripts originating distal to the infection site. Taken together, understanding how plant defense molecules are controlled at the cellular level requires new technological approaches.

Over the past decade, laser microdissection (LMD) has emerged as a robust way to isolate individual cells and tissues from complex organs and tissue systems (Day et al., 2005; Khan et al., 2014a; Gautam and Sarkar, 2015). Other technologies including fluorescently activated cell sorting and the isolation of nuclei tagged in specific cell types (INTACT) are limited in their applications due to the need for protoplasting or transformations with cell type-specific markers (Zhang et al., 2008; Deal and Henikoff, 2011). While there are variations in LMD design from different manufacturers, tissues are generally fixed and sectioned using common histological techniques and placed on specialized microscopy slides or plates. Depending on the system of study, wax or plastic can be used to embed tissues and preserve RNA (Inada and Wildermuth, 2005; Klink and Thibaudeau, 2014). Once fixed, the samples are then visualized using light or fluorescence microscopy and individual cells and tissues are selected and excised with a laser and collected for downstream molecular analysis (Schiebold et al., 2011; Gautam and Sarkar, 2015). The cellular-level resolution provided by LMD based tissue collection is therefore uniquely suited to overcome the often low coverage of pathogen transcripts and the signal dilution of pathogen-specific RNAs inherent to traditional RNA-seq experiments.

Laser microdissection has been used in model systems, and as a tool to discover how TFs are modulated in *Arabidopsis* leaves following infection by the biotrophic powdery mildew causing *Golovinomyces orontii* (Chandran et al., 2010). This seminal paper provided new insights into plant defense; however, the transcriptomic data were quantified using microarrays, a technology that relies on *a priori* knowledge of the system. In a similar array-based experiment, LMD was used to discover how different molecular processes occur in the spatially distinct infection regions of colonized poplar leaves (Hacquard et al., 2010) further supporting the application of the technique to fungal biology. Thus, LMD coupled with next generation RNA sequencing should detect a broader and more dynamic range of gene activity in addition to resolving new transcripts with essential roles in the regulation and integration of the plant defense process.

In the case of the complex tissue systems of the leaf, it is likely that each tissue or cell type plays a different or overlapping role in general cell function and plant defense. For example, when *Sclerotinia sclerotiorum* interacts with the canola leaf, the fungal hyphae first grow laterally along the leaf surface under the cuticle before penetrating the epidermis and moving through the mesophyll and finally infiltrating the vasculature leading to systemic colonization of the plant body. Therefore, the fungus is in direct contact with each type of tissue and investigating tissue-specific roles in defense will strengthen our understanding of plant defense systems.

Surprisingly, few plant mRNA profiling studies have used LMD to better understand the genetic response to pathogen interactions and none have combined this technology with a dual sequencing strategy, thus providing unprecedented opportunity for future research. However, these technologies have already provided insights into plant responses to nematodes in both tomato (Ramsay et al., 2004) and soybean (Klink et al., 2009), and pointed to modifications in sugar metabolism as a result of grapevine infection with a phytoplasma (Santi et al., 2013). The precision LMD adds to these experiments and makes it an essential tool for understanding the specific molecular events that influence the outcome of a host–pathogen interaction, and complement a broader strategy that utilizes genetic information to best overcome major crop pathogens.

## Computational Prediction of Biological Regulators

The majority of cellular reprogramming during the plant defense response is transcriptionally controlled through complex networks of TFs and their DNA binding sites (see Tsuda and Somssich, 2015, for review). The addition of dual sequencing and LMD to the plant pathologist’s tool kit will increase the resolution of these interactions, but does nothing to discover the transcriptional regulators of the genetic and molecular processes involved. Elucidating the complex regulatory network of TFs with their DNA binding sites and the sets of genes and biological processes they control will provide the foundation for building crops that are more resistant or tolerant to fungal pathogens. This complex task requires multiple resources encompassing data on experimentally and computationally derived TF – DNA binding site motif interactions in addition to annotated gene lists from co-expressed or differentially expressed gene sets (Belmonte et al., 2013; Khan et al., 2014b).

**FIGURE 2:**
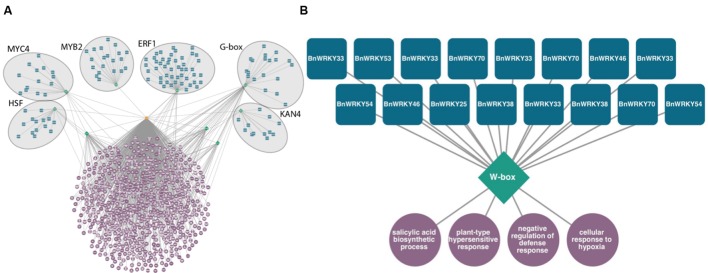
**Predicted transcriptional modules from sets of genes differentially expressed following fungal infection of *Brassica napus*.** Transcription factors (blue squircles) predicted to bind to DNA sequence motifs (green diamonds) located 1 kb upstream of transcription start sites of a set of genes (orange octagon) enriched in gene ontology terms (purple circles, *P* < 0.0001 hypergeometric distribution). **(A)** Transcriptional module within differentially expressed genes of *B. napus* in response to *Sclerotinia sclerotiorum*, Heatshock factor (HSF), MYC, MYB, Ethylene Response Factor (ERF), G-box, and KAN4 motifs are predicted to control defense related biological processes. **(B)** Predicted WRKY transcriptional circuit from genes specifically differentially expressed in *B. napus* resistant to *Leptosphaeria maculans* 3 days post-inoculation.

This type of tool can be used to discover potential transcriptional regulators in large sets of genes differentially expressed in response to fungal infection. For example, in *Sclerotinia*-infected canola leaves, six overrepresented DNA sequence promoter motifs, HSF, MYC4, MYB2, ERF1, G-box, and KAN4 are predicted to regulate genes associated with signaling, defense, and translation (**Figure 2A**). Likewise, smaller subsets of co-expressed gene sets can be analyzed (**Figure 2B**). These modules consisting of TFs predicted to bind to these DNA motifs found within gene sets are therefore potential regulators of these processes. Of genes exclusively differentially expressed in a resistant line of canola infected with *Leptosphaeria maculans*, 16 WRKY homologs are predicted to control genes associated with SA biosynthesis and the hypersensitive response (publicly available dataset available on NCBI’s Gene Expression Omnibus, GSE77723)^6^.

An opportunity also exists for the development of a similar prediction tool based on fungal sequencing data to better understand the regulation of processes involved in pathogenesis as well as an avenue to identify putative targets for functional applications. These bioinformatics tools can serve as a valuable resource to the scientific community through mining existing and previously published large scale genes expression data sets. Predicting transcriptional regulators in economically important crop pathogens using this targeted approach should allow researchers to identify genes essential for growth and pathogenesis quickly using functional tests.

## Protecting Crops with RNA Technology

Researchers are now able to apply transcriptomic data in the development of innovative crop protection technologies. RNA interference (RNAi), promises to best the current control broad spectrum measures, eliminate negative consequences of current disease control, and combat the alarming rise of fungicide resistant phytopathogens (Ishii and Holloman, 2015). RNAi specifically knocks down genes using an intrinsic cellular defense phenomenon. Through the detection and processing of double stranded RNA (dsRNA) or hairpin RNA (hpRNA) by fungal cells, transcripts are targeted using sequence homology leading to degradation or silencing (Nakayashiki, 2005). The application of cell specific and dual RNA sequencing data should provide the information to identify novel fungal targets. Hairpin RNA or dsRNA molecules can then be tailored for a specific transcript and upon delivery, can directly limit fungal pathogenesis.

Both dsRNA and hpRNA have the potential to protect cropping systems through topical applications or using a transgenic approach. Ghag et al. (2014) demonstrated the utility of transgenic plants expressing anti-pathogenicity RNA molecules against *Fusarium oxysporum*, the causative agent of *Fusarium* wilt. Banana plants were engineered to express intron hpRNA constructs for VELVET or FUSARIUM TRANSCRIPTION FACTOR 1 and maintained some level of resistance for at least 8 months. Despite the demonstrated success of RNAi technology against fungal pathogens, foliar applications have not yet come to fruition. However, they offer many benefits over transgenics including: the ability to explore a greater variety of novel targets compared to the production of stably transformed plants, a more rapid response to emerging pathogens and races, and wider public acceptance since host plant genomic changes have not occurred (Senthil-kumar and Mysore, 2010; Lucht, 2015). Fortunately, foliar application of RNAi technology has been successfully used as an insecticide in both lab and field studies (Baum et al., 2007; Whyard et al., 2009; Yu et al., 2013). In particular, San Miguel and Scott (2015) demonstrated the viability of a foliar application of actin dsRNA molecules to protect potato plants from Colorado potato beetles (*Leptinotarsa decemlineata*). The molecules were remarkably stable, showing bioactivity for over 28 days. With all the benefits and the proven viability of a topical application, future work should invest in the development of effective anti-fungal RNAi application methods.

In spite of the successes, some environmentalists are concerned with RNAi technology introducing large quantities of persistent molecules into the environment. Early results show dsRNA molecules will not persist or accumulate in soil (Dubelman et al., 2014). However, without a robust body of research on the environmental fate of RNA molecules, caution must be taken to prevent deleterious effects. Due to conserved sequences, molecules must be designed to have no more than 20 bases of homology to other transcripts, followed by thoroughly performing *in vitro* assays on various types of organisms. With meticulous molecular design, RNAi technology holds the promise to revolutionize agricultural disease management. While the cost to produce enough dsRNA molecules to protect broad acre crops is high, the expense to produce these molecules continues to decrease with the implementation of bacterial production systems (Palli, 2014; Robinson et al., 2014). The use of dsRNA molecules to protect against major crop pathogens will provide a targeted response for producers, and promises to be more effective while evading negative environmental consequences associated with broad spectrum fungicides.

## Outlook

The development of innovative research technologies to protect the agricultural landscape should provide the necessary tools to sustain global food demand. Through these technologies we have developed a deep understanding of host–pathogen interactions at the RNA level. However, there are still many gaps in our knowledge that surround emerging crop systems where genetic information is lacking. Fundamental details remain to be resolved on how plant defense and fungal pathogenic processes are specified at the cellular level at the site of infection and the contribution of transcriptional circuits controlling these processes. The application of RNA sequencing technologies coupled with cutting edge LMD methods should provide plant science researchers with answers to protect our food systems. While the analysis of large scale datasets still remains a challenge, new, and user friendly computational pipelines and programs will allow for broader access to and the potential for innovative product development. These strategies will also provide information essential for implementing the next generation of thorough, effective, and responsible RNAi-based fungal control measures in plant crop systems.

## Author Contributions

IG, AM, TK, DF, and MB conceptualized and wrote the paper. IG and MB drafted the figures.

## Conflict of Interest Statement

The authors declare that the research was conducted in the absence of any commercial or financial relationships that could be construed as a potential conflict of interest.

## Footnotes

1 araport.org

2 soybase.org

3 gramene.org

4 elbe.hki-jena.de/fungifun/

5 galaxyproject.org

6 http://www.ncbi.nlm.nih.gov/geo/
